# High flow nasal cannula for patients undergoing bronchoscopy and gastrointestinal endoscopy: A systematic review and meta-analysis

**DOI:** 10.3389/fsurg.2022.949614

**Published:** 2022-08-15

**Authors:** Yuan Tao, Mingyang Sun, Mengrong Miao, Yaqian Han, Yitian Yang, Xuhui Cong, Jiaqiang Zhang

**Affiliations:** Department of Anesthesiology and Perioperative Medicine, Zhengzhou University People's Hospital, Henan University People's Hospital, Henan Provincial People's Hospital, Zhengzhou, China

**Keywords:** high flow nasal cannula, hypoxemia, endoscopy, airway intervention, meta-analysis

## Abstract

**Background:**

High flow nasal cannula is gaining increasingly used in patients undergoing endoscopic procedures. We undertook this systematic review and meta-analysis to determine whether high flow nasal cannula (HFNC) could effectively minimize the risk of hypoxemia as compared with conventional oxygen therapy (COT).

**Methods:**

We performed a comprehensive search of Pubmed, Cochrane Central Register of Controlled Trials (CENTRAL), Embase, and Web of Science. Studies involving the application of HFNC during endoscopic procedures were identified.

**Results:**

We included 15 randomized controlled trials (7 bronchoscopy, 8 gastrointestinal endoscopy). Patients receiving HFNC during endoscopic procedures had a significantly lower risk of hypoxemia (defined as SpO_2_ < 90%) versus COT group (risk ratio = 0.32; 95%CI (0.22–0.47), 13 studies, 4,093 patients, moderate-quality evidence, *I*^2^ = 48.82%, *P* < 0.001). The lowest SpO_2_ was significantly higher in HFNC group (mean difference = 4.41; 95%CI (2.95–5.86), 9 studies, 1,449 patients, moderate-quality evidence, *I*^2^ = 81.17%, *P* < 0.001) than those receiving COT. No significant difference was detected between groups in end-procedure partial pressure of CO_2_ (standard mean difference  =  −0.18; 95%CI (−0.52–0.15), 5 studies, 238 patients, moderate-quality evidence, *I*^2^ = 42.25%, *P* = 0.29). Patients receiving HFNC were associated a lower need for airway intervention (risk ratio = 0.45; 95%CI (0.24–0.84), 8 studies, 2,872 patients, moderate-quality evidence, *I*^2 ^= 85.97%, *P* = 0.01) and less procedure interruption (risk ratio = 0.36; 95%CI (0.26–0.51), 6 studies, 1,562 patients, moderate-quality evidence, *I*^2^ = 0.00%, *P* < 0.001). The overall intubation rate after endoscopy was 0.20% in both group, with no difference detected (risk ratio = 1.00; 95%CI (0.30–3.35), 7 studies, 2,943 patients, low-quality evidence, *I*^2^ = 0.00%, *P* = 1.00).

**Conclusion:**

This systematic review and meta-analysis found moderate to low evidence that the application of HFNC was associated with improved oxygenation, decreased need for airway intervention, and reduced procedure interruption in patients undergoing endoscopic procedures. Future larger sample and high-quality studies are warranted to confirm our result and further investigate the effectiveness of HFNC in patients at risk.

**Systematic Review Registration**: https://www.crd.york.ac.uk/prospero/, identifier: CRD42022298032.

## Introduction

Endoscopic procedures are now widely performed for the diagnosis and treatment of many diseases in clinical practice. However, patient discomfort and intolerance may interrupt the successful implementation of endoscopy (1). Sedation helps to increase patient satisfaction and facilitate clinical manipulation, especially in prolonged procedures or technically demanding therapeutic interventions (2). It's estimated that over half of the endoscopic procedures are now performed under monitored anesthesia with sedation (3, 4). Meanwhile, sedation may render the patient unconscious and unable to protect the airway, increasing the risk of hypoxemia and other cardiopulmonary complications (5, 6). More importantly, during certain procedures such as endoscopic retrograde cholangiopancreatography (ERCP), it is difficult for the anesthesiologist to get access to the patient's airway because the endoscopic device has occupied the oral cavity. Therefore, it is essential to provide supplemental oxygenation and maintain the airway stable during endoscopic procedures.

Several techniques have been introduced during the endoscopic procedures, with conventional oxygen therapy (COT) as the most widely used device, usually including low flow nasal cannula, nasopharyngeal tube, and simple face mask. But COT could only provide oxygen flow up to 15 L/min and a limited oxygen concentration of 30%–50%, on account of the air mixing and dilution from dead space (7, 8). Supraglottic jet ventilation and non-invasive ventilation (NIV) have also been implemented as valid alternatives to minimize desaturation (9, 10). However, patient discomfort and the difficulty of manipulating the endoscopy through the mask preclude its widespread application. As a recently developed novel technique, high flow nasal cannula (HFNC) delivers rather high flow (maximum 70 L/min), heated and humidified gas (31–37 °C) with adjustable oxygen concentration (21%–100%) through a dedicated nasal cannula (11, 12). As compared with COT and NIV, HFNC could better match patients’ respiratory demands without complex settings. The efficiency and safety of HFNC in the intensive care unit and operating room have been demonstrated recently (13, 14). Nonetheless, the utility of HFNC during endoscopic procedures is still controversial and remains to be determined (15, 16). The purpose of this systematic review aims to compare the incidence of hypoxemia, lowest oxygen saturation, partial pressure of carbon dioxide (CO_2_) at the end of the procedure, airway intervention, procedure interruption, and intubation rate after procedure in patients undergoing endoscopic procedures compared to conventional oxygen therapy.

## Methods

### Study protocol and registration

The protocol of this review was conducted in accordance with the Preferred Reporting Items for Systematic Review and Meta-Analysis Protocols (PRISMA-P) checklist and was registered with PROSPERO (CRD: 42022298032). Given that all study data had been previously published and this review didn’t include any individual patient data, no institutional review board approval was required.

### Study selection, inclusion and exclusion criteria

We identified randomized controlled trials that compared HFNC with COT in patients undergoing endoscopic procedures. We excluded articles that focus on the following population: (1) patients < 17 years old, (2) pregnancy, (3) undergoing bronchoscopy for intubation, (4) duplicate patient cohorts, and (5) published as reviews or case reports.

### Search strategy

We performed a comprehensive search of PubMed, Cochrane Central Register of Controlled Trials, Web of Science, and Embase from inception to April 10 in 2022. The search strategy concept blocks were built on the topics of (high flow nasal cannula) AND (endoscopic) AND (RCTs), limited to human, and no language restrictions were imposed. The search included the combination of the following Medical Subject Headings (MeSH): “high flow nasal cannula”, “high flow nasal oxygen”, “nasal high flow”, “HFNC”, “HFNO”, “NHF”, “Thrive”, “Optiflow”, “endoscopy”, “gastroscopy”, “colonoscopy”, “bronchoscopy”, and “endoscopic retrograde cholangiopancreatography”. The detailed search strategy was displayed in Supplementary Material S1.

Study characteristics were extracted by two independent investigators (YT and MS) throughout the screening process with a predefined data collection form. The data extracted included the following information: author, year of publication, sample size, population characteristics, sedation techniques, and intervention settings. The outcomes extracted were: incidence of hypoxemia (defined as SpO_2_ < 90%), lowest oxygen saturation, partial pressure of carbon dioxide (CO_2_) at the end of procedure (including end-tidal CO_2_ (EtCO_2_), and arterial blood gas CO_2_), airway intervention (defined as chin lift, head tilt, jaw thrust, insertion of oral/nasal airway, and mask ventilation), procedure interruption and intubation rate after procedure. We also checked the supplementary data and contacted the authors for more detailed information if necessary. Any divergence was determined by reaching a consensus or consulting a third reviewer (MM).

### Quality assessment and publication bias

We assessed the methodological quality of the included studies using the revised Cochrane Collaboration Risk of Bias (RoB 2) tool, consisting of five different domains: bias arising from the randomization, bias due to deviation from intended interventions, bias due to missing outcome data, bias in the measurement of the outcome, and bias in the selection of the reported data (17). And each domain was rated as low risk, some concern, or high risk. Two reviewers (YH and YY) independently made the RoB 2 judgment and disagreements were resolved by discussion in the presence of a third reviewer (XC). The guidelines of the Recommendations Assessment, Development, and Evaluation (GRADE) system were applied to evaluate the level of certainty for the results (18). The main contents included: risk of bias, inconsistency, indirectness, imprecision, and publication bias. The level of certainty was graded using GRADEpro version 3.6 software.

### Statistical analysis

Statistical analysis was performed with Stata 16.0 by an independent statistician. Dichotomous outcomes (hypoxemia, airway intervention, procedure interruption, and intubation rate after procedure) were presented as frequency and proportion, and pooled risk ratio (RR) with 95% confidence interval (CI) was estimated by a random-effects model (DerSimonian-Laird method). For continuous outcomes (lowest oxygen saturation, partial pressure CO_2_ at the end of procedure), we calculated pooled estimates of the mean difference (MD) or standard mean difference (SMD) and 95%CI with a random-effects model. In some trials, mean and standard deviations (SD) were estimated from the provided median and interquartile range (IQR) (19, 20). Statistical heterogeneity and the inconsistency of treatment effects across studies were evaluated using *I*^2^ statistics, and were divided into the following three levels: low (*I*² < 50%), moderate (*I*² = 50%–75%), and high (*I*² > 75%) (21). Funnel plots were used to assess the possibility of publication bias and Egger regression test was used to measure funnel plot asymmetry. Duval and Tweedie's trim and fill analysis was also performed to further evaluate the potential effects of publication bias.

We planned the following prespecified subgroup analyses when studies were enough (no less than 2 studies): (1) bronchoscopy versus gastrointestinal endoscopy, (2) HFNC with high FiO_2_ (FiO_2_ > 50%) versus HFNC with low FiO_2_ (FiO_2_ ≤ 50%), (3) high-risk population (fulfilling one of the following criterion: lung-transplant recipients, American Society of Anesthesiologists (ASA) physical status 3 or 4, obesity (BMI ≥ 30 kg/m^2^), known or suspected obstructive sleep apnea (OSA)) versus low-risk population, (4) procedures with different level of sedation (2).

## Results

### Search results and study characteristics

**Figure 1:**
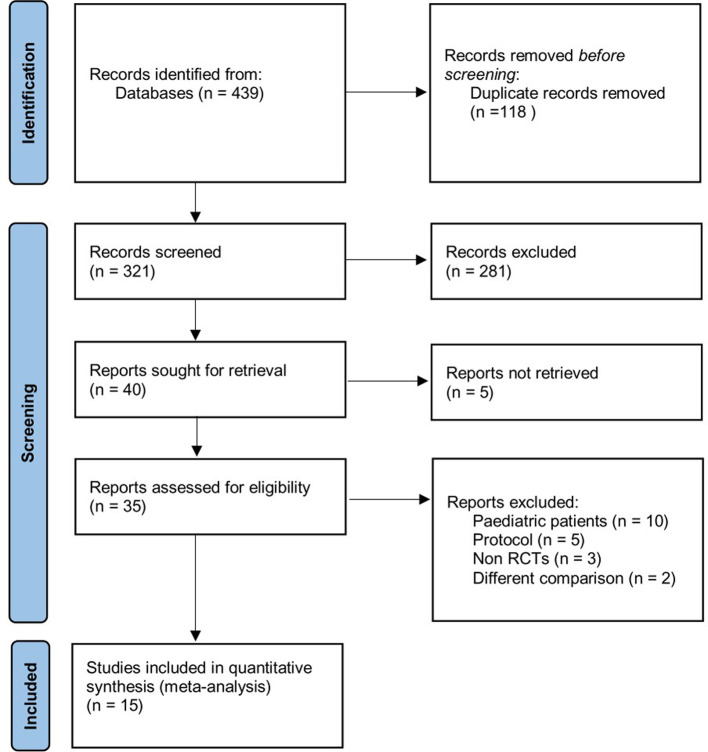
PRISMA flow diagram.

Of the 439 citations identified through our initial search, 118 were duplicates. After the screening through the title and abstract, 281 were excluded. 40 studies were assessed for eligibility. Full-text articles excluded were pediatric patients, protocols, studies with abstract only, studies without COT as a comparator, and non-RCT studies. Finally, a total of 15 RCTs (4,451 patients) were pooled into our final quantitative analysis from 2012–2021 (15, 16, 22–34), as shown in Figure 1. Table 1 presents detailed information of these trials.

**Table 1:** Main characteristics of included studies.

Author (year)	Number of patients	Patient characteristics	Type of procedure	Procedure Duration (min)	Sedatives	Degree of sedation	HFNC settings	COT settings	Outcomes reported
Ben-Menachem 2020 (22)	76	Age ≥ 18, ASA II–III, Lung-transplant recipients	Bronchoscopy	HFNC:33.0 COT:34.0	Midazolam Propofol Alfentanil	Deep sedation	Flow:30–50 L/min FiO_2_: unclear	Flow: 4–10 L/min through nasal cannula	Hypoxemia Lowest SpO_2_ Airway intervention Procedure interruption
Douglas 2017 (23)	60	Age ≥ 18, ASA I–IV	Endobronchial ultrasound Bronchoscopy	HFNC:24.0 COT:21.0	Midazolam Opioids Propofol	Conscious sedation	Flow:30–70 L/min (median 50 L/min) FiO_2_:100%	Flow: 10–15 L/min through bite block	Hypoxemia Lowest SpO_2_ End procedure ETCO_2_ Airway intervention
Ifran 2020 (24)	40	Age ≥ 18, Baseline SpO_2_ ≥ 90%	Endobronchial ultrasound Bronchoscopy	Not reported	Midazolam Alfentanil	Conscious sedation	Flow:70 L/min FiO_2_:100%	Flow: 2 L/min through nasal prongs	Hypoxemia Lowest SpO_2_ End procedure ETCO_2_ Intubation rate
Kim 2021 (25)	72	Age ≥ 20, ASA I–IV	ERCP	HFNC:17.5 COT:15.3	Propofol Fentanyl	Deep sedation	Flow:50 L/min FiO_2_:100%	Flow: 5 L/min through nasal cannula	Hypoxemia Lowest SpO_2_ End procedure ETCO_2_ Airway intervention Procedure interruption
Lee 2021 (26)	187	Age ≥ 65, ASA I–III	ERCP	HFNC:17.0 COT:16.0	Propofol Midazolam	Deep sedation	Flow:50 L/min FiO_2_:50%	Flow: 5 L/min through nasal cannula	Hypoxemia Lowest SpO_2_ Procedure interruption
Lin 2019 (16)	1994	Age ≥ 18, ASA I–II	Gastroscopy	HFNC:5.0 COT:5.1	Propofol	Deep sedation	Flow:60 L/min FiO_2_:100%	Flow: 2 L/min through nasal cannula	Hypoxemia Airway intervention Intubation rate
Longhini 2021 (27)	36	Age ≥ 18	Bronchoscopy	HFNC:11.5 COT:12.8	No sedatives	No sedation	Flow:60 L/min FiO_2_:21%	Flow: up to 6 L/min through nasal cannula	Hypoxemia Lowest SpO_2_ End procedure PaCO_2_ Intubation rate
Lucangelo 2012 (28)	45	Age ≥ 18, BMI < 30, Baseline SpO_2_ ≥ 90%	Bronchoscopy	HFNC60:15.0 HFNC40:15.0 COT:14.0	Midazolam	Conscious sedation	HFNC60: Flow:60 L/min FiO_2_:50% HFNCN40: Flow:40 L/min FiO_2_:50%	Flow: 40 L/min through Venturi mask	End procedure PaO_2_ End procedure PaCO_2_
Mazzeffi 2020 (29)	262	Age > 17	Advanced esophagogastro-duodenoscopy	HFNC:32.0 COT:30.2	Propofol Fentanyl Midazolam	Deep sedation	Flow:20 L/min FiO_2_: unclear	Flow: 6 L/min through nasal cannula	Hypoxemia SpO_2 _<_ _92% Peak TcPCO_2_ Intubation rate
Nay 2021 (30)	379	Age ≥ 18, ASA I–IV, moderate or high risk of hypoxemia	Gastrointestinal endoscopy	HFNC:21.0 COT:19.0	Propofol	Deep sedation	Flow:70 L/min FiO_2_:50%	Flow: 5–6 L/min through nasal cannula or face mask or nasopharyngeal tube	Hypoxemia Airway intervention Procedure interruption Intubation rate
Riccio 2019 (15)	59	Age 18–80, ASA II–IV BMI > 40	Colonoscopy	HFNC:33.0 COT:30.5	Lidocaine Propofol	Conscious to deep sedation	Flow: up to 60 L/min FiO_2_:36–40%	Flow: 4 L/min through nasal cannula	Hypoxemia Lowest SpO_2_ Airway intervention
Teng 2019 (31)	152	Age 20–80, ASA I–II, Baseline SpO_2_ ≥ 90%	Esophagogastro-duodenoscopy	HFNC:6.0 COT: 5.8	Midazolam Alfentanil Propofol	Deep sedation	Flow:30 L/min FiO_2_:100%	Flow: 5 L/min through nasal cannula	Hypoxemia Airway intervention Intubation rate
Thiruvenkatarajan 2021 (32)	131	Age > 18, Fulfilling one of the following: ASA ≥ III, BMI > 30, known or suspected OSA	ERCP	Not reported	Propofol Fentanyl	Deep sedation	Flow: up to 60 L/min FiO_2_:100%	Flow: 8 L/min through nasal cannula + mouthguard	Hypoxemia Lowest SpO_2_ Peak TcPCO_2_ Airway intervention Intubation rate
Ucar 2021 (33)	170	Age ≥ 18, BMI ≤ 30, Baseline SpO_2_ ≥ 90%	Endobronchial ultrasound Bronchoscopy	HFNC:18.0 COT: 18.0	Midazolam	Unclear level	Flow: 35 L/min FiO_2_:40%	Flow: 5 L/min through nasal cannula	Hypoxemia
Wang 2021 (34)	788	Age > 18, Baseline SpO_2_ ≥ 90%	Bronchoscopy	HFNC:11.4 COT: 13.3	No sedatives	No sedation	HFNC: 50 L/min FiO_2_: up to 36%	Flow: up to 6 L/min through nasal prongs	Hypoxemia Lowest SpO_2_ Procedure interruption

HFNC, high flow nasal cannula; COT, conventional oxygen therapy; OSA, obstructive sleep apnea; BMI, Body mass index; ERCP, endoscopic retrograde cholangiopancreatography; ETCO_2_, end-tidal partial pressure of CO_2_; TcPCO_2_, percutaneous partial pressure of CO_2_.

The included studies covered a wide range of situations in the endoscopic procedures, including bronchoscopy (22, 27, 28, 34), endobronchial ultrasound (EBUS) (23, 24, 33), endoscopic retrograde cholangiopancreatography (ERCP) (25, 26, 32), esophagogastroduodenoscopy (16, 29–31), and colonoscopy (15). The settings of HFNC ranged differently, with the flow from 20 L/min–70 L/min, and inspired oxygenation from 21%–100%. With regard to the devices utilized in the conventional oxygen therapy group, most were nasal cannulas, and the remaining included venturi mask, bite block oxygen insufflation, and nasopharyngeal tube. Sedation techniques included in this systematic review comprised deep sedation, conscious sedation, and no sedation, with most studies (7 RCTs) using a deep level of sedation (2).

### Hypoxemia during the procedure

**Figure 2:**
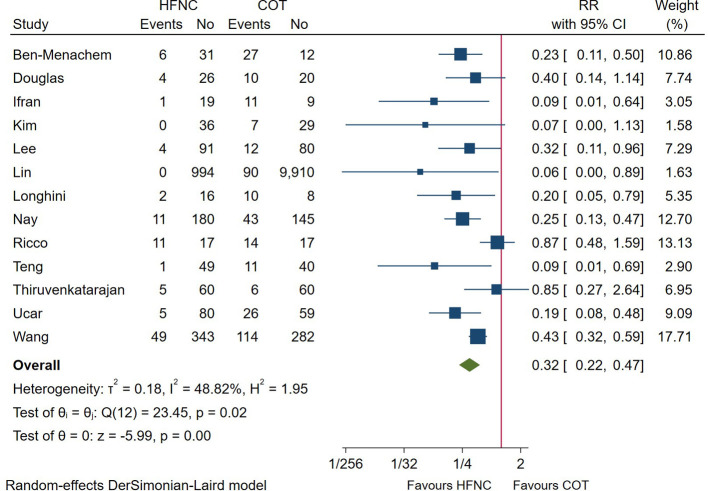
Forest plot comparing hypoxemia during the procedure in HFNC versus COT. HFNC, high flow nasal cannula; COT, conventional oxygen therapy; RR, risk ratio.

A total of 14 trials reported the incidence of hypoxemia during the endoscopic procedure, with 13 studies meeting our prespecified primary outcome (defined as a SpO_2_ < 90%). As shown in Figure 2, the incidence of hypoxemia was significantly lower in patients receiving HFNC as compared with those receiving COT (RR = 0.32; 95%CI (0.22–0.47), 13 studies, 4,093 patients, moderate-quality evidence, *I*^2^ = 48.82%, *P* < 0.001). Considering the heterogeneity of included studies, we divided the studies according to the sedation technique. Pooled results showed that patients receiving deep sedation also showed a reduced incidence of hypoxemia (RR = 0.27; 95%CI (0.17–0.43), *I*^2^ = 19.30%), while no difference was detected in those receiving conscious sedation (RR = 0.43; 95%CI (0.15–1.23), *I*^2^ = 64.40%), as displayed in Supplementary Figure S1A. Other predefined subgroup analysis regarding FiO_2_, procedure, and risk of the patient didn’t alter the result.

### Lowest SpO_2_ during the procedure

**Figure 3:**
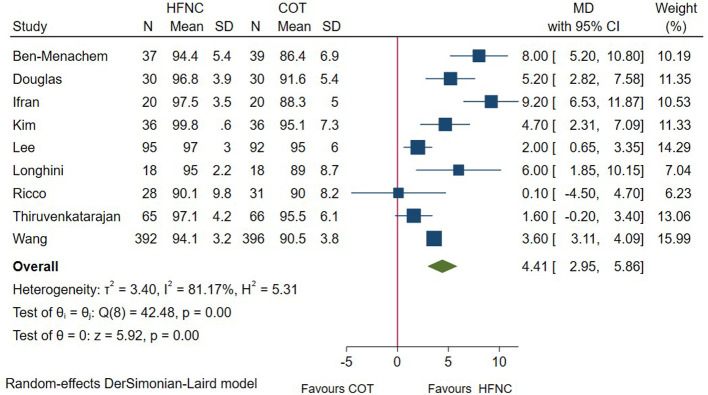
Forest plot comparing lowest SpO2 during the procedure in HFNC versus COT. HFNC, high flow nasal cannula; COT, conventional oxygen therapy; MD, mean difference.

In total, 9 RCTs measured the lowest SpO_2_ in patients undergoing endoscopic procedures. Pooled results showed that the minimum SpO_2_ during the procedure was significantly higher in HFNC group (MD = 4.41; 95%CI (2.95–5.86), 9 studies, 1,449 patients, moderate-quality evidence, *I*^2^ = 81.17%, *P* < 0.001) versus the conventional oxygen therapy group (Figure 3). However, subgroup analysis showed the influence of risk of patient on this outcome (high-risk patient: MD = 3.38; 95%CI (−1.35–8.11), *I*^2^ = 87.70%). (Supplementary Figure S1B). Regarding other subgroup analysis, our result remained unchanged.

### End-procedure partial pressure of CO_2_

**Figure 4:**
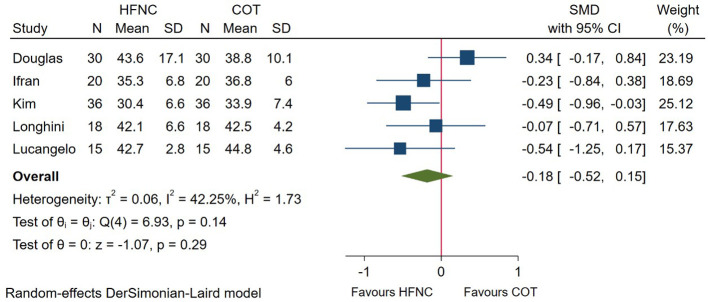
Forest plot comparing end-procedure partial pressure of CO2 during the procedure in HFNC versus COT. HFNC, high flow nasal cannula; COT, conventional oxygen therapy; SMD, standard mean difference.

During the procedure, partial pressure of CO_2_ was measured through arterial blood gas analysis (PaCO_2_), end-tidal CO_2_ monitoring (ETCO_2_), or percutaneous CO_2_ monitoring (TcPCO_2_). Among these, 5 studies reported the end procedure partial pressure of CO_2_. Pooled data (Figure 4) showed that no significant difference was detected between HFNC and COT group (SMD = −0.18; 95%CI (−0.52–0.15), 5 studies, 238 patients, moderate-quality evidence, *I*^2^ = 42.25%, *P* = 0.29). In addition, subgroup analysis showed that the result was consistent whether measured through PaCO_2_ (SMD = −0.28; 95%CI (−0.76–0.21), *I*^2^ = 40.50%) or ETCO_2_ (SMD = −0.13; 95%CI (−0.64–0.38), *I*^2^ = 64.50%), as displayed in Supplementary Figure S1B.

### Airway intervention during the procedure

**Figure 5:**
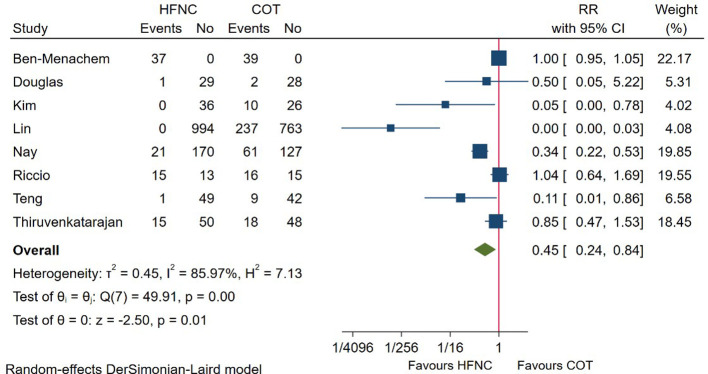
Forest plot comparing airway intervention during the procedure in HFNC versus COT. HFNC, high flow nasal cannula; COT, conventional oxygen therapy; RR, risk ratio.

In total, 8 RCTs reported the incidence of airway intervention, including chin lift, head tilt, jaw thrust, insertion of oral/nasal airway, and mask ventilation. Meta-analysis based on these studies showed that patients receiving HFNC required less airway intervention (RR = 0.45; 95%CI (0.24–0.84), 8 studies, 2,872 patients, moderate-quality evidence, *I*^2^ = 85.97%, *P* = 0.01) as compared with those receiving COT (Figure 5). However, subgroup analysis showed that no difference was detected between groups in high-risk patient (RR = 0.75, 95%CI (0.45–1.23), *I*^2^ = 86.30%) (Supplementary Figure S1A), and those receiving HFNC with a FiO_2_ ≤ 50% (RR = 0.59; 95%CI (0.20–1.77), *I*^2^ = 90.90%). In addition, the beneficial effect of HFNC no longer existed during bronchoscopy (RR = 1.00; 95%CI (0.95–1.05), *I*^2^ = 0.00%) (Supplementary Figure S1A).

### Procedure interruption

**Figure 6:**
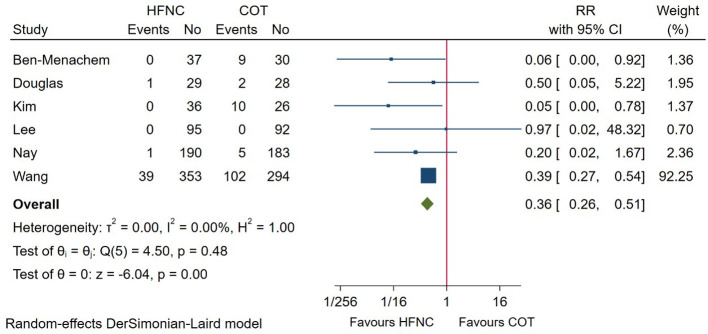
Forest plot comparing procedure interruption during the procedure in HFNC versus COT. HFNC, high flow nasal cannula; COT, conventional oxygen therapy; RR, risk ratio.

6 studies compared the incidence of procedure interruption between HFNC and COT. Pooled evidence in Figure 6 showed that procedure interruption was significantly lower among patients receiving HFNC versus COT (RR = 0.36; 95%CI (0.26–0.51), 6 studies, 1,562 patients, moderate-quality evidence, *I*^2^ = 0.00%, *P* < 0.001). Subgroup analysis based on procedure or risk of patient didn’t alter the result (Supplementary Figure S1A).

### Incidence of intubation after the procedure

**Figure 7:**
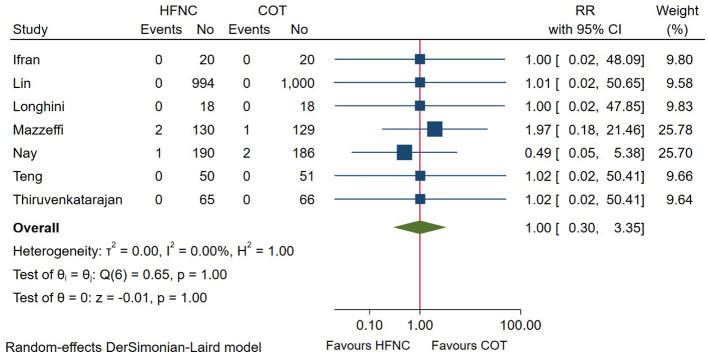
Forest plot comparing incidence of intubation after the procedure in HFNC versus COT. HFNC, high flow nasal cannula; COT, conventional oxygen therapy; RR, risk ratio.

7 RCTs reported the incidence of intubation after the endoscopic procedure. Pooled quantitative analysis showed that the intubation rate after endoscopy was 0.20% (3/1,470) in the HFNC group and 0.20% (3/1,473) in the COT group, with no difference detected between groups (RR = 1.00; 95%CI (0.30–3.35), 7 studies, 2,943 patients, low-quality evidence, *I*^2^ = 0.00%, *P* = 1.00), as displayed in Figure 7. Considering the low number of events, no subgroup was performed as meaningful conclusions would not be possible.

### Sensitivity analysis and publication bias

**Figure 8:**
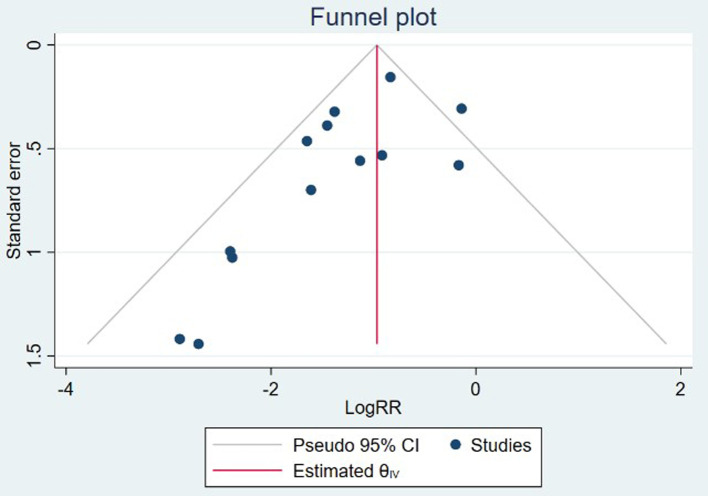
Funnel plot for publication bias.

Sensitivity analysis was performed by excluding one study each time from the included studies. The leave-one-out sensitivity analysis indicated that the study conducted by Douglas et al. (23) might be the source of heterogeneity, by omitting which resulted in a lower level of CO_2_ (SMD = −0.35; 95%CI (−0.64–−0.06), *I*^2^ = 0.00%), as shown in Supplementary Figure S2. In addition, after removing the trial conducted by Lin et al. (16), no significant difference was found in terms of the airway intervention (RR = 0.61; 95%CI (0.37–1.03), *I*^2^ = 80.70%) between groups (Supplementary Figure S3). Other results of our study were robust (Supplementary Figures S4–S7). Publication bias was detected by the visual inspection of the funnel plots (Figure 8) and further confirmed by the Egger regression test (*z* = −2.42, *P* = 0.02). The trim and fill analysis of funnel plot for the primary outcome indicated up to 4 unreported trials (Supplementary Figure S8).

### Risk of bias and GRADE evidence quality

**Figure 9:**
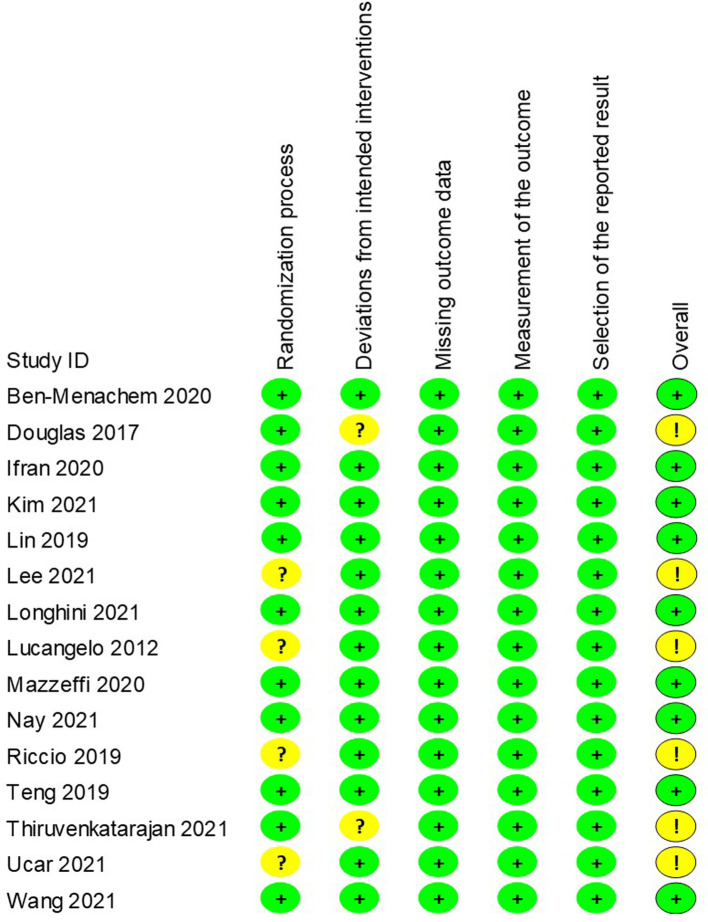
Revised risk of bias of randomised controlled trials. Green circle, low risk; yellow circle, some concerns; red circle, high risk.

The revised risks of bias assessment of the included studies were shown in Figure 9. Four of the studies were rated as some concerns in the domain of randomization (15, 26, 28, 33), and another two were rated as some concerns regarding deviation from intended intervention (23, 32). The GRADE evidence quality for the main results was summarized in Table 2. The quality of evidence was low for the incidence of intubation after the procedure, moderate for hypoxemia, lowest SpO_2_, airway intervention, procedure interruption, and end procedure partial pressure of CO_2_.

**Table 2:** GRADE evidence quality.

Outcome	No. of studies	Risk of bias	Inconsistency	Indirectness	Imprecision	Publication bias	Effect size (95%CI)	Certainty	Importance
Hypoxemia	13	not serious	not serious	not serious	not serious	strongly suspected^a^	RR: 0.32 (0.22, 0.47)	⊕⊕⊕⊖ moderate	Critical
Lowest SpO_2_	9	not serious	not serious	not serious	not serious	strongly suspected^a^	MD: 4.41 (2.95, 5.86)	⊕⊕⊕⊖ moderate	Critical
End-procedure CO_2_	5	not serious	not serious	not serious	not serious	strongly suspected^a^	SMD: −0.18 (−0.52, 0.15)	⊕⊕⊕⊖ moderate	Critical
Airway intervention	8	not serious	not serious	not serious	not serious	strongly suspected^a^	RR: 0.45 (0.24, 0.84)	⊕⊕⊕⊖ moderate	Critical
Procedure interruption	6	not serious	not serious	not serious	not serious	strongly suspected^a^	RR: 0.36 (0.26, 0.51)	⊕⊕⊕⊖ moderate	Critical
Intubation	7	not serious	not serious	not serious	serious^b^	strongly suspected^a^	RR: 1.00 (0.30, 3.35)	⊕⊕⊖⊖ low	Critical

^a^ Data reported as downgraded because of detected publication bias.

^b^ Data reported as downgraded because of few events in both group and wide confidence interval.

## Discussion

This systematic review provided a moderate-to-low level of certainty that the application of HFNC was associated with decreased incidence of desaturation, airway intervention, and procedure discontinuation, as well as improved SpO_2_ in patients undergoing endoscopic procedures. Concerning the level of end-procedure CO_2_ and intubation rate after the procedure, no significant difference was detected.

The potential mechanisms of hypoxemia in patients receiving sedatives consist of reduced respiratory drive, decreased laryngeal muscle tone, ventilation/perfusion (V/Q) mismatch, and transformed breathing pattern (35). During bronchoscopy, gas aspiration-related atelectasis, increased airway resistance, and bronchoalveolar lavage may further lead to the deterioration of gas exchange (28). HFNC delivers oxygen flow up to 70 L/min, which matches or exceeds the patient's spontaneous inspiratory flow, thus decreasing the entrainment of ambient air during inspiration and maintaining a stable FiO_2_. Besides, HFNC generates a flow-dependent positive airway pressure and increases the end-expiratory lung volume, therefore helping to overcome airway resistance and keep the airway open (36). Another physiological effect of HFNC is that the heated and humidified gas helps to improve mucociliary clearance and patient comfort (37). More importantly, except for the easy initiating and simplicity of intra-procedure management, HFNC allows enough airway space for manipulation, which is of vital importance during bronchoscopy and upper gastrointestinal procedures.

Several previous systematic reviews have demonstrated the effect of HFNC during the endoscopic procedure. Su et al. (38) investigated the application of HFNC in patients undergoing bronchoscopy and concluded that HFNC could reduce the incidence of hypoxemic events and improve oxygenation. However, all of their included studies had a small sample size with a total of 257 patients (5 RCTs), and no subgroup analysis was performed due to the lack of sufficient data. Hung et al. (39) conducted a meta-analysis of applying HFNC in sedated patients receiving gastrointestinal endoscopic procedures. Similarly, their pooled estimates found that HFNC revealed lower risks of hypoxemia, airway interventions, procedural interruption, and a lower level of CO_2_. In their subgroup analysis based on age, oxygen flow, gender, risk status, and type of procedure, the beneficial effects of HFNC were consistent in these clinical settings. Spence and colleagues (40) identified studies involving intraoperative use of HFNO in surgical patients, and finally included 8 RCTs (4 during induction, 4 during procedure, 2,314 patients). Their study showed that the application of HFNC was associated with prolonged safe apnea time during induction of general anesthesia and better oxygenation during endoscopic procedures. Our review incorporated the most recent studies (15 RCTs and 4,451 patients) with strict inclusion criteria and well-defined subgroup analysis, thus further demonstrating the effectiveness and safety of HFNC in endoscopy procedures.

The settings of HFNC differed among these RCTs and may partly explain the source of heterogeneity. Some designs of the included studies provided a FiO_2_ of 100% to patients in the HFNC group (16, 23–25, 31, 32). For these receiving conventional oxygen, 2 to 10 L/min oxygen *via* nasal cannula or face mask was administered, providing a FiO_2_ of less than 50% on average. To isolate the PEEP or dead-space washout effects from the elevated inspired oxygen concentration, some recent studies chose a lower FiO_2_ to provide a similar FiO_2_ as the COT group (15, 30, 33, 34). Accordingly, we conducted subgroup analysis to further investigate the influence of FiO_2_ on the results. Pooled estimate demonstrated that patients receiving lower FiO_2_ were also associated with a reduced risk of hypoxemia, less procedure interruption, and improved lowest SpO_2_, although the effect size was reduced. Therefore, our findings suggested that the PEEP and dead-space washout effects of HFNC play a critical role in preventing desaturation, in addition to the effect of elevated FiO_2_.

Sedation techniques implemented during the endoscopic procedure also varied widely, which may result in different extent of respiratory-related complications. Therefore, we divided the included studies into the following sedation level: no sedation, conscious sedation, and deep sedation (2). Our subgroup analysis indicated that firm evidence was reached in people receiving deep sedation. Notably, in those under conscious sedation, the strength of HFNC was no longer observed, which suggested that people receiving a deep level of sedation may benefit more from HFNC. Consistent with our result, Spence et al. (40) proved the effectiveness of HFNC in improving oxygenation and prolonging the safe apnea time during induction of general anesthesia (40). Another major concern that may confound the result was the heterogeneous population in our meta-analysis. Some studies enrolled high-risk patients, including lung-transplant recipients, obesity (BMI > 30 kg/m^2^), known or suspected OSA, and high ASA physical status, while others were relatively low-risk patients. Therefore, we performed a subgroup analysis accordingly and found that HFNC could also help to decrease the incidence of desaturation in high-risk patients. However, in terms of other secondary outcomes (airway intervention and lowest SpO_2_), no difference was detected. Therefore, future larger studies are warranted to investigate the effectiveness and safety in patients with high-risk factors.

Despite the proven clearance effect of HFNC on CO_2_, the pooled estimate didn’t show a reduced partial pressure of CO_2_ at the end of endoscopy, which was consistent with the previously published meta-analysis (40). The potential explanation was that most of the procedures were conducted under deep sedation, which led to some extent of respiratory suppression and apnea. Under this circumstance, rising PetCO_2_ and acidosis may be unavoidable. In addition, some patients (present with CO_2_ retention) are prone to hypoventilation while receiving increased inspired oxygenation fraction, because of alterations in hypoxic pulmonary vasoconstriction and physiologic dead space (23, 41). Therefore, it's necessary to strengthen monitoring and shorten the endoscopy duration when applying HFNC, especially in vulnerable patients (such as obesity, OSA, and COPD).

The complications of HFNC were reported in some RCTs, including nasopharyngeal dryness, itching from oxygen, and abdominal bloating (31, 32). Considering the scarcity of the available data and heterogeneous reported outcomes, it was not plausible to pool the results together. But most of them were self-limited and no increasement in medical management was required. More importantly, the increased tolerance in patients receiving HFNC may reduce the incidence of agitation and decrease the frequency of associated severe complications (for instance, pneumothorax) (34).

Our study has several limitations. Firstly, heterogeneity in most analyses was high, which may possibly be due to types of procedure, patient characteristics, HFNC settings, and sedation technique. Notwithstanding, we divided the patients accordingly to explore more detailed subgroup effects. Secondly, we didn’t examine patient or proceduralist satisfaction, considering that the definitions varied and the results were inconsistently reported. Thirdly, the pre-registered protocol was violated during our implementation. We initially intended to include endoscopic procedures with sedation only, but we subsequently decided to enroll all types of study and further analyzed the sedation technique utilized as a part of the subgroup analysis. Lastly, publication bias was detected among the included studies, and the result should be interpreted with caution.

## Conclusion

Our systematic review and meta-analysis indicated that the application of HFNC was associated with improved oxygenation, decreased need for airway intervention, and reduced procedure interruption in patients undergoing endoscopic procedures. We suggest the utilization of HFNC for endoscopic procedures, especially in those receiving deep sedation. Further studies comparing HFNC and conventional oxygen therapy in patients at risk of hypoxemia should be performed in endoscopic settings.

## Data Availability

The original contributions presented in the study are included in the article/**Supplementary Material**, further inquiries can be directed to the corresponding author/s.
