# Supplementary material for: High flow nasal cannula for patients undergoing bronchoscopy and gastrointestinal endoscopy: A systematic review and meta-analysis
Source: Front Surg. 2022 Aug 15;9:949614. doi: 10.3389/fsurg.2022.949614 (PMC9420969; doi:10.3389/fsurg.2022.949614)

Supplementary Figure 1. Subgroup analysis of the comparison between groups.

Supplementary Figure 2 Sensitivity analysis of removing the study by Douglas et al. in terms of end procedural CO_2_.


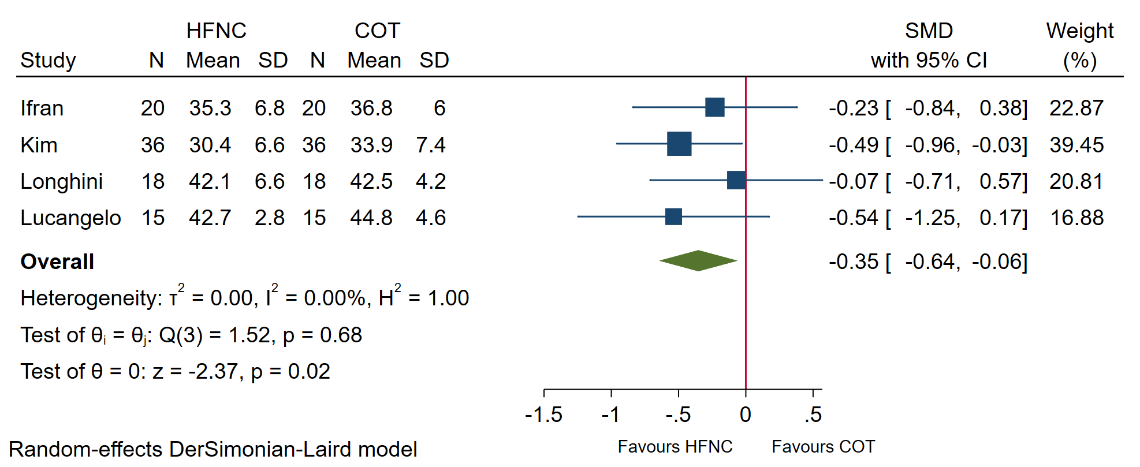


Supplementary Figure 3 Sensitivity analysis of removing the study by Lin et al. in terms of the airway intervention.


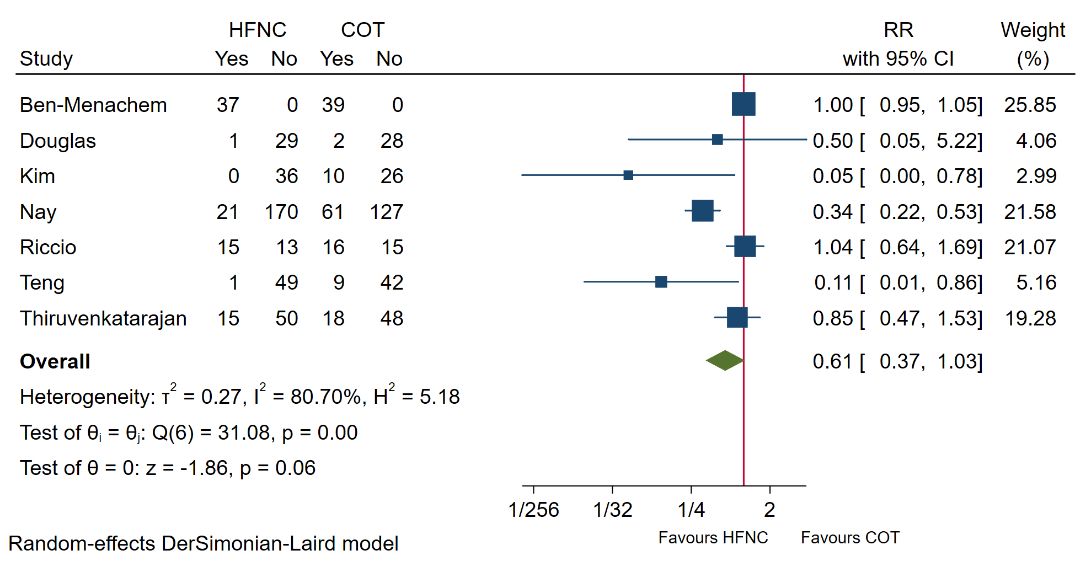


Supplementary Figure 4 Sensitivity analysis of hypoxemia during the procedure.


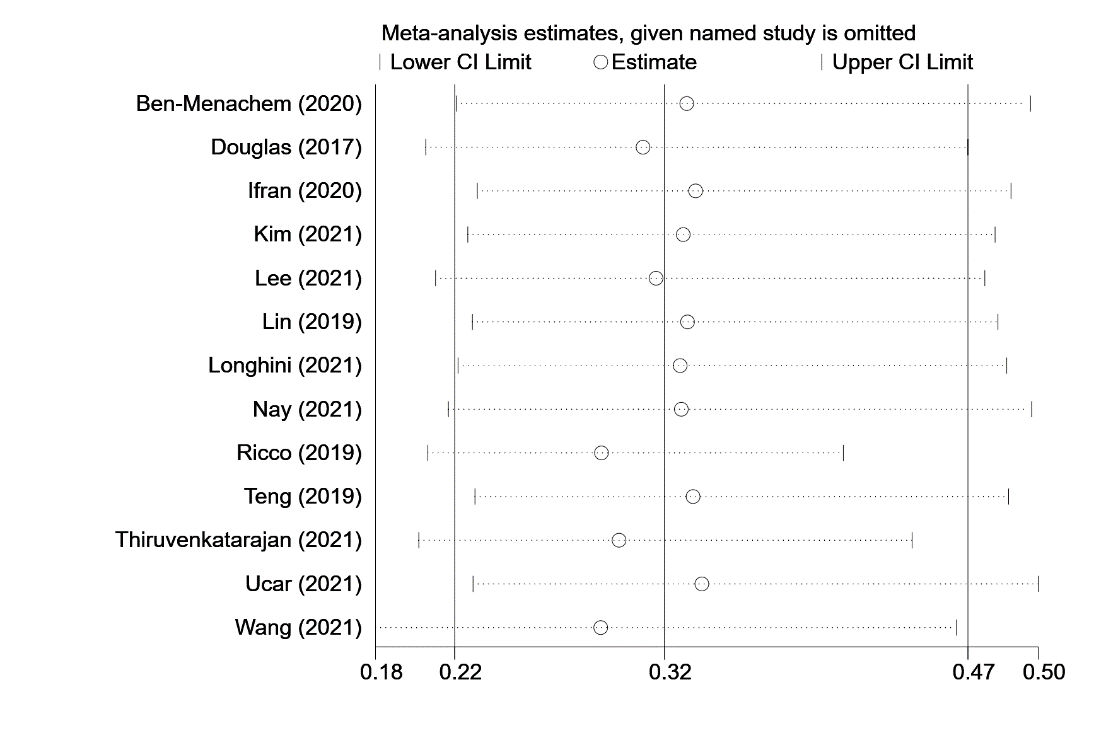


Supplementary Figure 5 Sensitivity analysis of lowest SpO_2_ during the procedure.


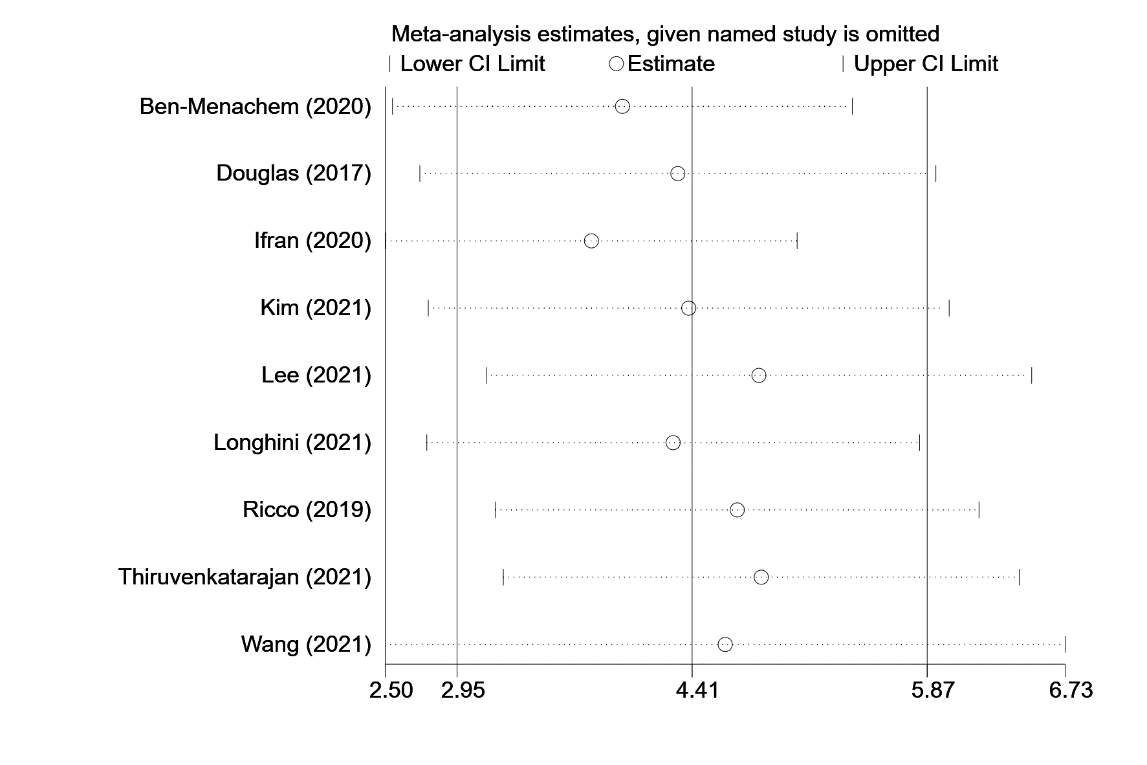


Supplementary Figure 6 Sensitivity analysis of procedure interruption .


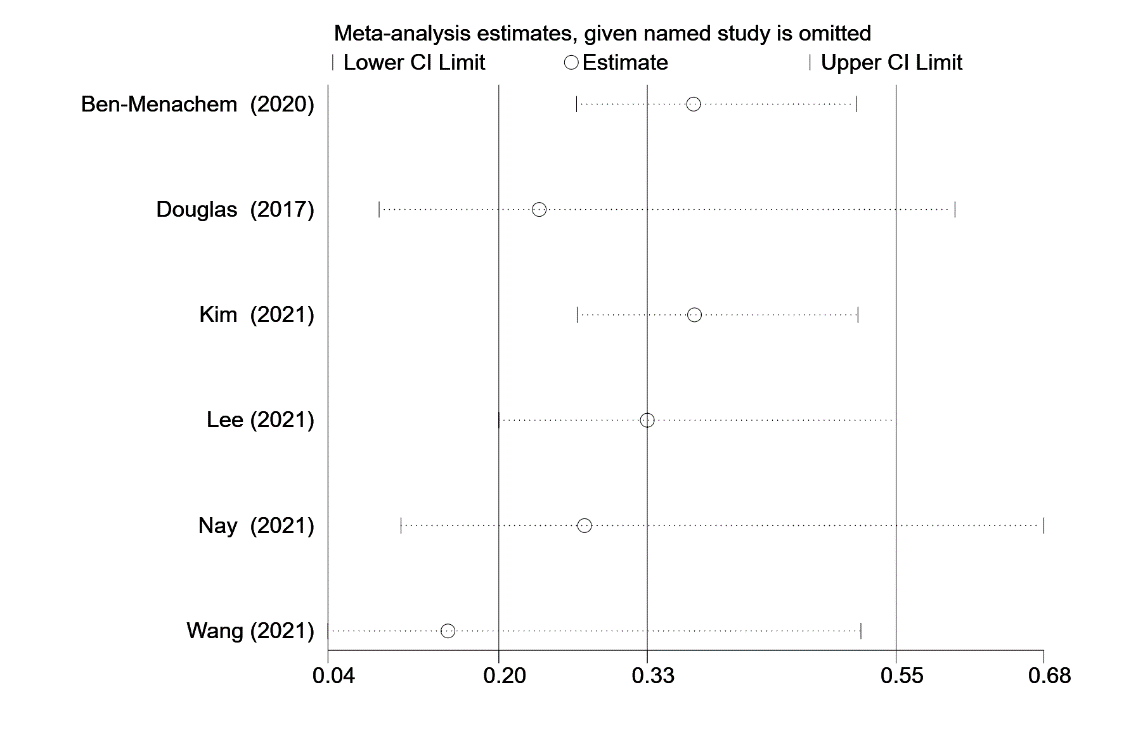


Supplementary Figure 7 Sensitivity analysis of incidence of intubation after the procedure.


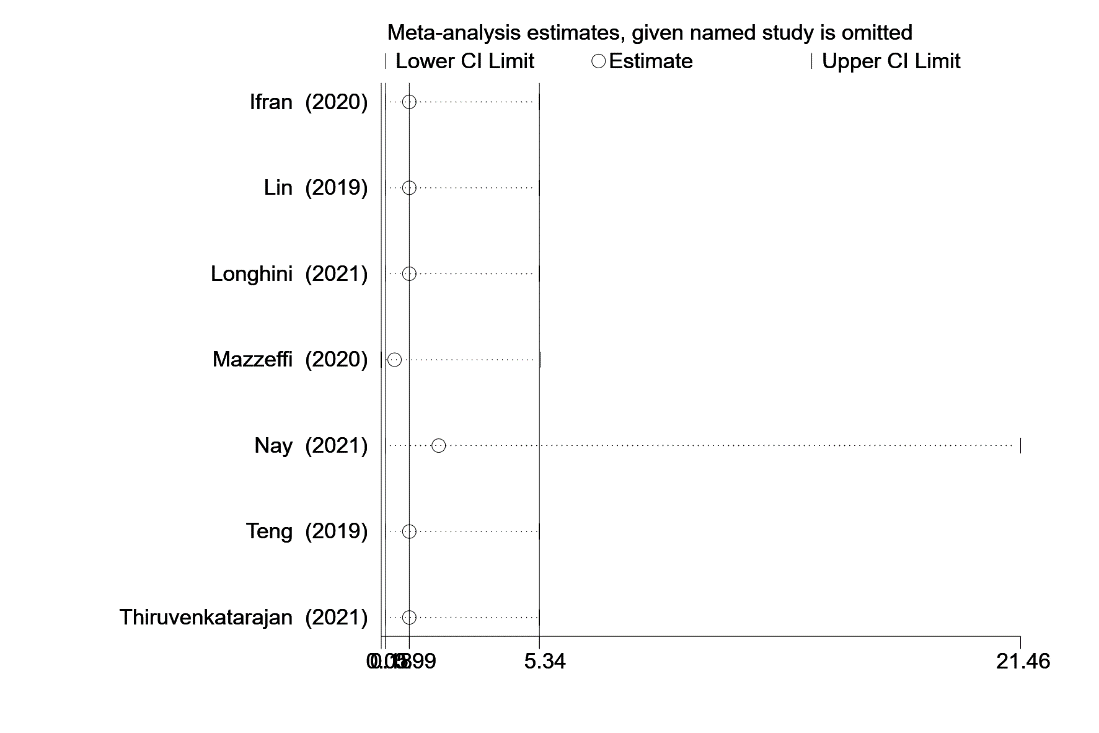


Supplementary Figure 8 The trim and fill analysis of funnel plot.


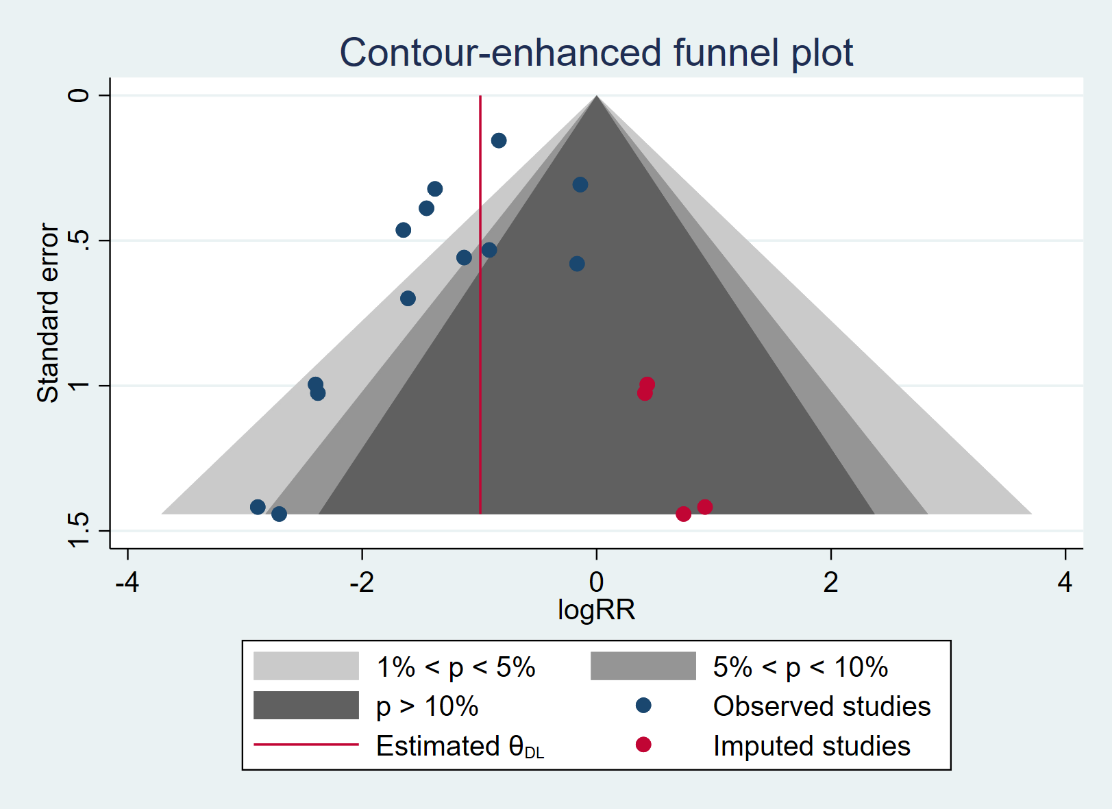

Supplement: Supplementary file 1 [file Data_Sheet_1_v1.docx]
